# Healthy Neonates Possess a CD56-Negative NK Cell Population with Reduced Anti-Viral Activity

**DOI:** 10.1371/journal.pone.0067700

**Published:** 2013-06-21

**Authors:** Amanda Jacobson, Frank Bell, Nicholas Lejarcegui, Caroline Mitchell, Lisa Frenkel, Helen Horton

**Affiliations:** 1 Seattle Biomedical Research Institute, Seattle, Washington, United States of America,; 2 Seattle Children's Hospital, Seattle, Washington, United States of America; 3 Departments of Pediatrics, University of Washington, Seattle, Washington, United States of America; 4 Department of Medicine, University of Washington, Seattle, Washington, United States of America; 5 Department of Obstetrics & Gynecology, University of Washington, Seattle, Washington, United States of America; 6 Department of Lab Medicine, University of Washington, Seattle, Washington, United States of America; 7 Department of Global Health, University of Washington, Seattle, Washington, United States of America; Karolinska Institutet, Sweden

## Abstract

**Background:**

Neonatal Natural Killer (NK) cells show functional impairment and expansion of a CD56 negative population of uncertain significance.

**Methods:**

NK cells were isolated from cord blood and from adult donors. NK subpopulations were identified as positive or negative for the expression of CD56 and characterized for expression of granzyme B and surface markers by multi-parameter flow cytometry. Cell function was assessed by viral suppression and cytokine production using autologous lymphocytes infected with HIV. Activating (NKp30, NKp46) and inhibitory (Siglec-7) markers in healthy infants and adults were compared with viremic HIV-infected adults.

**Results:**

Cord blood contained increased frequencies of CD56 negative (CD56neg) NK cells with reduced expression of granzyme B and reduced production of IFNγ and the CC-class chemokines RANTES, MIP1α and MIP1β upon stimulation. Both CD56pos and CD56neg NK subpopulations showed impaired viral suppression in cord blood, with impairment most marked in the CD56neg subset. CD56neg NK cells from cord blood and HIV-infected adults shared decreased inhibitory and activating receptor expression when compared with CD56pos cells.

**Conclusions:**

CD56neg NK cells are increased in number in normal infants and these effectors show reduced anti-viral activity. Like the expanded CD56neg population described in HIV-infected adults, these NK cells demonstrate functional impairments which may reflect inadequate development or activation.

## Introduction

Natural killer (NK) cells are important effectors in the early response to infection and there is increasing recognition of their role as regulators of the adaptive immune response in addition to the containment of infection through cytokine production and the killing of infected cells [1], [2]. Infants localize and contain infectious agents poorly [3], and infectious diseases continue to claim responsibility for the majority of annual global infant deaths [4]. Infants with vertically-transmitted HIV infection, including those infected after birth through exposure to maternal breast-milk [5], have poor rates of survival and high viral loads compared with individuals infected later in life [6], [7]. NK cells from umbilical cord blood consistently demonstrate poor cytotoxic function and generate reduced quantities of IFNγ and other cytokines when compared with NK cells obtained from adults (reviewed in references [8] & [9]). However, there are conflicting data regarding granzyme B expression in neonatal NK cells. One study has shown decreased levels of granzyme B, which may contribute to the impaired cytotoxic ability of cord blood NK cells [10], while others report levels of lytic effectors similar to those found in adults [11], [12].

There is increasing awareness that NK cells are a heterogeneous population with different receptor expression and different functional profiles [13], [14]. Despite this, the vast majority of previous studies in neonates have focused on bulk NK cell responses or have limited analysis to cells expressing CD56. Unlike adult peripheral blood, umbilical cord blood contains a significant proportion of CD56negCD16pos cells [11], [15], [16], hereafter referred to as ‘CD56neg’, a subpopulation of NK cells also described in individuals with chronic viral infections [17], including hepatitis C [18]–[20] and HIV [21]–[23] infection. Similar CD56neg NK populations have been reported following hematopoietic cell [24], including cord blood [25], transplantation. The CD56neg NK cells described from these varied settings have uniformly poor cytolytic ability with impaired cytokine production, characteristics that have led investigators to describe them as ‘immature’ [11], dysfunctional' [23] or ‘anergic’ [14]. However, as is true for cord blood CD56pos NK cells, cytolytic function in cord blood CD56neg cells appears to be rapidly restored by incubation with IL-2, IL-12 or IL-15 [11], suggesting that functional disturbances in this newborn effector population may instead reflect an environment in which NK cells receive inadequate stimulation from dendritic cells, or alternatively, are unable to respond to physiological levels of signaling.

CD56neg NK cells from viremic HIV-infected adults display altered patterns of inhibitory and activating receptors, with decreased expression of natural cytotoxicity receptors NKp30 and NKp46 together with an increase in inhibitory receptors such as LIR (ILT2) [22]. Interestingly, an increase in the proportion of CD56neg NK cells was not identified in a study of HIV-infected children, although these authors describe changes in NK degranulation and receptor expression [26]. Few studies have examined cord blood NK cells in the control of HIV replication [27] and none have assessed this specific CD56neg NK subset or cytokine production in response to HIV-infected cells. In addition, bulk NK cell populations in cord blood typically demonstrate increased expression of NKp30 and NKp46, with decreased levels of LIR [9], [28]–[30], but to our knowledge no published studies have assessed receptor expression in CD56neg subpopulations in cord blood.

This study aimed to further characterize the population of cord blood CD56neg NK cells from healthy newborn infants by comparing the ability of CD56neg and CD56pos NK cells to control viral replication in autologous, HIV-infected CD4 lymphocytes and to investigate patterns of associated cytokine production. In addition, since this ‘aberrant’ population of CD56neg cells has been described in adults with chronic viral infections, we compared the expression of activating and inhibitory receptors among CD56neg cells from cord blood and from HIV-infected individuals.

Our data show that NK cells from cord blood demonstrate reduced suppression of viral replication in autologous cells infected with HIV *in vitro* when compared with NK cells obtained from healthy adults. This functional impairment particularly affects CD56neg cells, an NK subpopulation expanded in the newborn and found to demonstrate altered NK receptor expression with changes in the pattern of cytokine production on challenge with HIV-infected cells.

## Methods

### Ethics Statement

The Institutional Review Boards at the University of Washington and Seattle BioMedical Research Institute approved the study. All adults provided written informed consent. Cord blood specimens were obtained without linkage to maternal records and were deemed not to represent Human Subject Research. Consent for the use of these samples was not required by the Institutional Review Boards.

### Subjects, Sample Collection and Processing

Adult blood samples were collected from healthy, HIV-seronegative volunteers at the Seattle Biomedical Research Institute. Cord blood was collected from healthy, full-term infants born by scheduled Caesarean section prior to the onset of labor at the University of Washington, Department of Obstetrics and Gynecology, using sodium-heparin vacutainers. Cord blood mononuclear cells (CBMC) or adult PBMC were isolated over Ficoll-Hypaque gradients and cryopreserved. PBMC from HIV viremic individuals were obtained through the Center for AIDS Research (CFAR) at the University of Washington. The viremic group was composed of HIV-1 infected individuals that had been HIV-positive for greater than 2 years who were anti-retroviral therapy naïve with HIV viral loads of greater than 10 000 copies/µl.

### Flow Cytometry and FACS Sorting

Natural killer cells obtained from healthy adult and umbilical cord blood samples were identified and subpopulations characterized using antibody conjugates against CD3, CD14, CD33 and CD19 (all on FITC), CD16 (PE), CD56 (APC), granzyme B (AF 700), and acquired using a BD LSR II ™ flow cytometer (Becton Dickinson, Franklin Lakes NJ, USA). Antibody-fluorophore conjugates were obtained from BD Biosciences (San Jose CA, USA). Viability was determined using Live Dead Aqua (Invitrogen, Eugene, OR, USA). Cells were gated to exclude populations expressing CD3 (T lymphocytes), CD14 (monocytes), CD33 (myeloid cells & precursors) or CD19 (B lymphocytes). Staining against granzyme B was carried out after cell membrane permeabilization using FACS Permeabilizing solution 2 (BD Biosciences, San Jose CA, USA). For all experiments comparing expression levels of receptors by flow cytometry samples were thawed, stained and acquired together on the cytometer on the same day, using the same voltage settings. Data were analyzed using FlowJo software (Tree Star, Ashland OR, USA).

Comparison of activating and inhibitory surface markers among NK cells from healthy cord samples, healthy adults and viremic HIV-infected adults were obtained using a similar panel with antibodies to CD3, CD14, CD33 & CD19 (on FITC), CD16 (PE), CD56 (APC), with either CD328 (Siglec-7)/biotin (via streptavidin/BV605) and NKp30 (PE Cy5) or NKp46 (PE Cy7).

For viral suppression assays, NK cells from healthy cord and adult donors were sorted to CD56posCD16pos (‘CD56pos’) and CD56negCD16pos (‘CD56neg’) subpopulations using BD FACSAria II (Becton Dickinson) using fluorophores conjugated with CD3 (APC), CD14 (PE), CD16 (PE Cy7) & CD56 (PE Cy5). Viability was determined using Live Dead Violet (Invitrogen), and CD3 and CD14 were used to exclude T cells and monocytes. Post-sort purities were assessed and CD56pos or CD56neg NK cell fractions were routinely ≥90% (data not shown). These NK subpopulations were used as effectors in the viral suppression assays.

### Viral Suppression and Luminex Assays

Target cells for the viral suppression assay were generated by enriching (negative selection) for CD4pos T cells using magnetic bead isolation (EasySep, StemCell, Vancouver BC, Canada). CD4pos T cells were cultured at 2x10^6^ cells/ml in complete RPMI containing PHA and 10 U/ml IL-2 for 3 days. On day 3, CD4pos T cells were infected with HIV-LAI or HIV-CSF_JR_ at an MOI of 0.01 using Viromag magnetofection (OZ Biosciences, Marseille, France) and cultured in 96 well plates with 50 U/ml IL-2 with or without the addition of autologous isolated CD56pos or CD56neg NK cells at effector : target (E∶T) ratios of 10∶1, 5∶1, or 1∶1. Between 20,000–40,000 infected CD4 target cells and 20,000–250,000 NK effectors were used in the assays. On day 3, 5, and 7 post co-culture, 100 µl of the supernatants were collected and stored at −80°C and refreshed with 100 µl of complete RPMI + IL-2 for the remainder of the assay. HIV replication was assessed by production of p24 in supernatants and quantified using p24 ELISA (PerkinElmer, Waltham MA, USA). Viral suppression was determined by levels of p24 with effector NK cells and infected target CD4 cells relative to control infected target cells alone. Uninfected CD4 T cells were also cultured in parallel as a negative control.

Supernatants from the viral suppression assay on day 3 were thawed at room temperature and briefly spun to remove cellular debris. The Luminex assay was performed on a single run using the 18-plex Milliplex MAP kit (Millipore, Billerica MA, USA). Cytokines and chemokines measured were IFNγ, MIP1α, MIP1β, RANTES, IL-13, IL-6, TNFα, IL-8, GM-CSF, IL-10, IL-12p40, IL-12p70, IL-15, INFα2, sIL-2Rα, IL-1β, IP-10, and MCP1. Assays were read using the Luminex 200 (Invitrogen) and analyses performed using Excel.

### Statistical Analyses

Statistical analysis was performed with Prism version 5.0 (GraphPad, La Jolla CA, USA) using the non-parametric Mann-Whitney U test. Analyses of variance or equivalent non-parametric tests were used for initial evaluation of continuous data from several groups. Where these analyses suggested significant differences between groups, differences were explored by pair-wise comparison. For comparison of viral suppression data for NK cells from adult and cord blood we used generalized estimating equations with an exchangeable correlation matrix to account for potential correlation due to repeated measures from the same subject. All p values were two-sided and considered significant if less than or equal to 0.05.

## Results

### Increased Frequency of CD56negCD16 pos NK Cells in Healthy Neonates Compared With Healthy Adults

**Figure 1:**
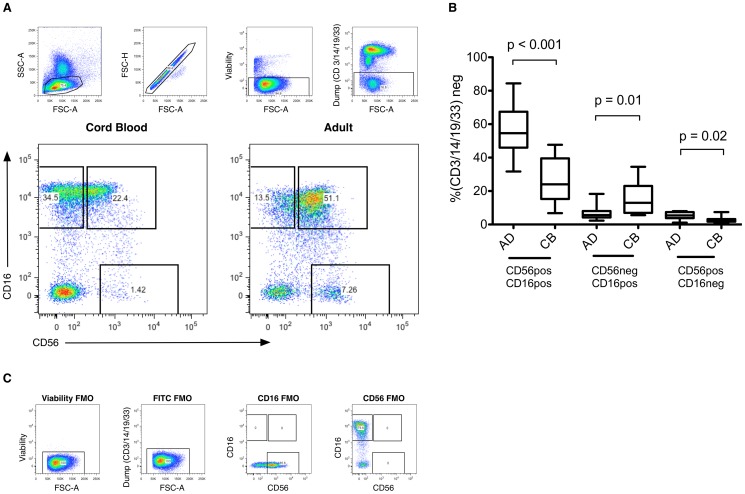
NK Cell Subpopulations in Cord Blood and Adult Peripheral Blood from Healthy Subjects. (A) Identification of NK cells from stained mononuclear cell populations was accomplished by selecting the live, singlet, lymphocyte population. Cells were gated to exclude expression of CD3 (T lymphocytes), CD14 (monocytes), CD19 (B lymphocytes) or CD33 (myeloid cells & precursors), and further characterized by CD56 and CD16 surface expression. (B) Proportions of major NK cell subpopulations present in umbilical cord blood (CB) (n = 10) and adult peripheral blood (AD) (n = 10). Box plots represent median and interquartile range (box) with data limits (whiskers). Data were compared between groups using the Mann-Whitney test. (C) Fluorescence Minus One (FMO) plots employed in generating the data displayed.

Comparing the frequency of NK cell subpopulations as determined by CD16 and CD56 staining (Figure 1A), a higher proportion of CD56negCD16pos NK cells were present in cord blood compared with adult blood (p = 0.01; Figure 1B). The proportions of CD56posCD16pos NK cells (also described as ‘CD56dim’) and of CD56posCD16neg NK cells (‘CD56bright’) showed corresponding reductions in cord blood compared with adults (p<0.001 and 0.02 for CD56posCD16pos and CD56posCD16neg respectively; Figure 1B).

### Neonatal NK Cells Have Decreased Expression of Granzyme B

**Figure 2:**
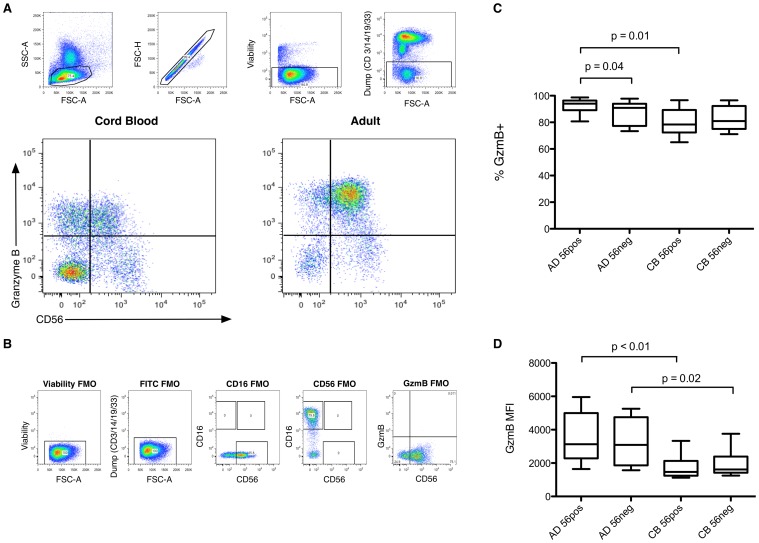
Granzyme B Expression in NK Cell Subpopulations from Cord Blood and Healthy Adult Subjects. (A) Representative plots showing granzyme B against CD56 expression for NK cells from adult and cord blood donors. (B) Fluorescence Minus One (FMO) plots employed in generating the data displayed. (C) Proportion of cells positive for granzyme B (GzmB+) among NK cell subpopulations isolated from cord blood (CB) (n = 10) and adult peripheral blood (AD) (n = 10). NK cells were defined as CD56 positive (56pos) or negative (56neg) and stained for granzyme B with expression assessed by flow cytometry. (D) Levels of granzyme B expression as measured by mean fluorescence intensity (MFI) in cord blood and in adult NK cell subpopulations. NK cells were identified from mononuclear cells by selecting live singlets from the lymphocyte population, gated to exclude CD3 (T lymphocytes), CD14 (monocytes), CD19 (B lymphocytes) or CD33 (myeloid cells & precursors), and characterized by CD56 and CD16 surface expression. Box plots indicate median and interquartile range (box) with data limits (whiskers). Data comparisons were performed between groups using the Mann-Whitney test.

FMO data for granzyme B analysis and examples of the flow cytometry staining data are shown in Figure 2 A and B). Among CD56pos NK cells, the frequency of cells expressing granzyme B was lower in cord blood compared with adult samples (p = 0.01; Figure 2C), and among all NK cells positive for this key lytic effector, the expression of granzyme B as measured by mean fluorescence intensity was lower in NK subsets from cord blood compared with adults (p<0.01 and 0.02 for CD56pos and CD56neg cells respectively; Figure 2D). In adult samples, fewer CD56neg NK cells expressed granzyme B than CD56pos cells (p = 0.04, Figure 2C), but no significant differences were seen between NK subpopulations derived from cord blood.

### Phenotypic Profiles of Neonatal NK Cells Compared With HIV-Viremic and Healthy Adult NK Cells

**Figure 3:**
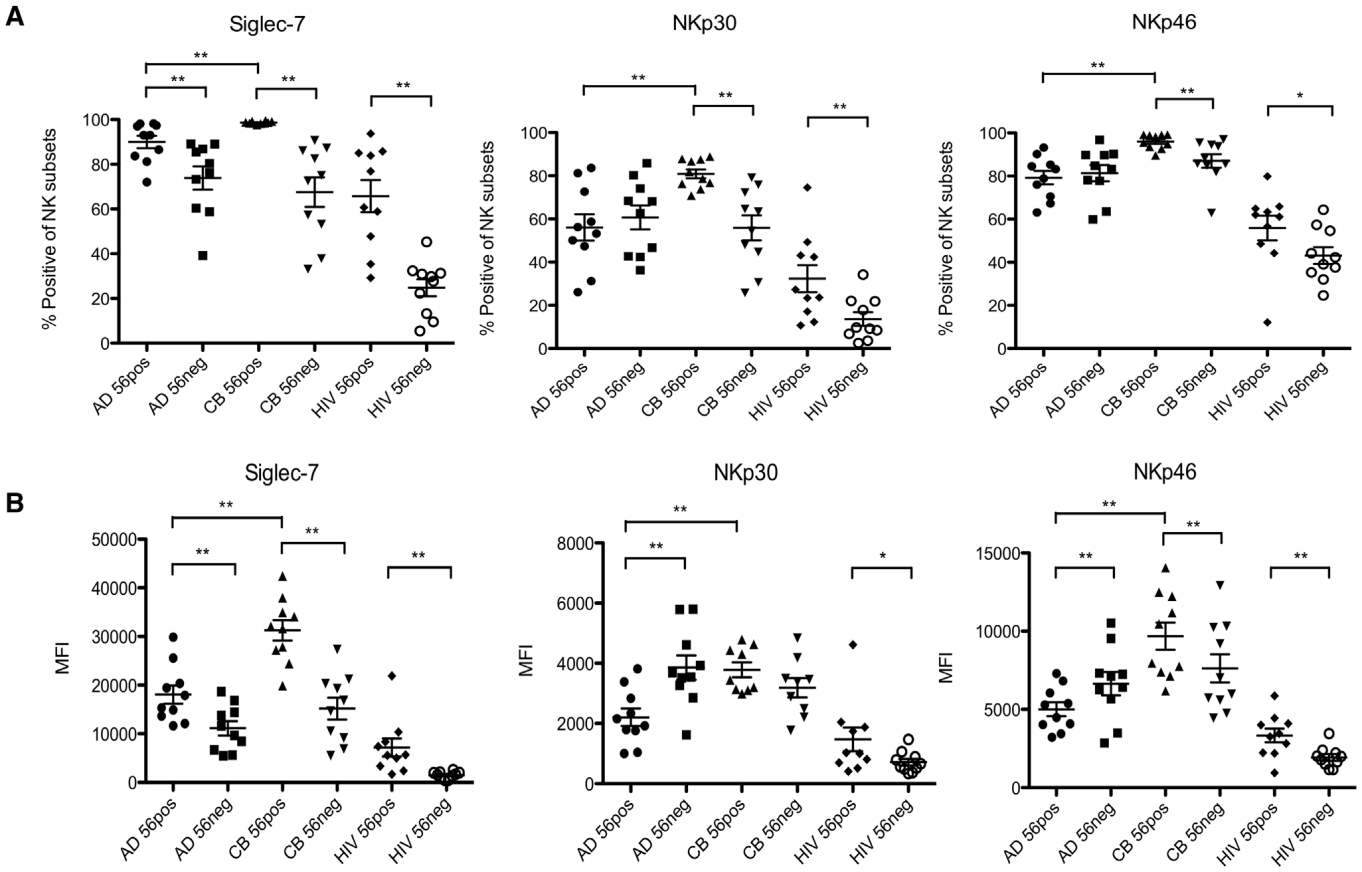
Expression of NK Receptors among NK Cell Subpopulations from Cord Blood, HIV-Infected and Healthy Adults. Surface NK markers were assessed by flow cytometry on CD56posCD16pos (56pos) and CD56negCD16pos (56neg) NK cell subpopulations from cord blood (CB) (n = 10), healthy adult HIV-seronegative individuals (AD) (n = 10) and viremic HIV-infected donors (HIV) (n = 10). A) Proportion of NK subsets staining positive for each receptor. B) Mean fluorescence intensity (MFI) for receptor expression among NK subpopulations positive for each surface receptor. Data between groups were compared using the Mann-Whitney test, * p<0.05, ** p<0.01. Bars represent mean ± SEM. Siglec  =  sialic acid binding immunoglobulin-like lectin. Representative plots showing receptor expression together with the Fluorescence Minus One (FMO) plots employed in these analyses are provided in Figure S1.

Because a CD56neg NK cell population has also been described in viremic HIV-infected individuals, we compared the phenotype of NK cells from HIV-viremic individuals and from healthy neonates. NK cells from healthy adults, HIV-viremic adults and cord blood from healthy infants were assessed for surface markers previously reported to be modified in HIV infection [23], [31]. Expression levels of surface receptors NKp30, NKp46 and Siglec-7 among CD56pos and CD56neg populations from each group are shown as the frequency of cells positive for each receptor in Figure 3A and as the mean fluorescence intensity in Figure 3B. FMO data and examples of the flow cytometry staining for each receptor are shown in Figure S1. Compared to CD56pos NK cells, CD56neg cells from HIV-viremic individuals expressed decreased levels of the activating receptors NKp46 and NKp30 (p<0.05 for NKp46, p<0.01for NKp30, Figure 3A & 3B), together with decreased expression of the inhibitory receptor Siglec-7 (p<0.01). Neonatal CD56neg NK cells demonstrated a similar pattern, with significant differences in expression between neonatal CD56pos and CD56neg populations for NKp46, NKp30, and Siglec-7 (p all <0.01).

CD56neg cells make up a relatively minor population in healthy adults and only limited phenotypic data have previously been described for these NK cells [18]. Of the CD56neg cells we identified from healthy adults we noted a reduction in inhibitory Siglec-7 expression (p<0.01) (Figure 3A & 3B).

CD56pos NK cells from cord blood expressed greater levels of the activating cytotoxic receptors NKp30 and NKp46, with higher levels of the inhibitory receptor Siglec-7 compared with CD56pos effectors from adult blood (p all <0.01for the proportions of CD56pos NK cells positive for all three receptors; Figure 3A & 3B).

### Neonatal NK Cells Have Decreased Ability to Control HIV Replication

**Figure 4:**
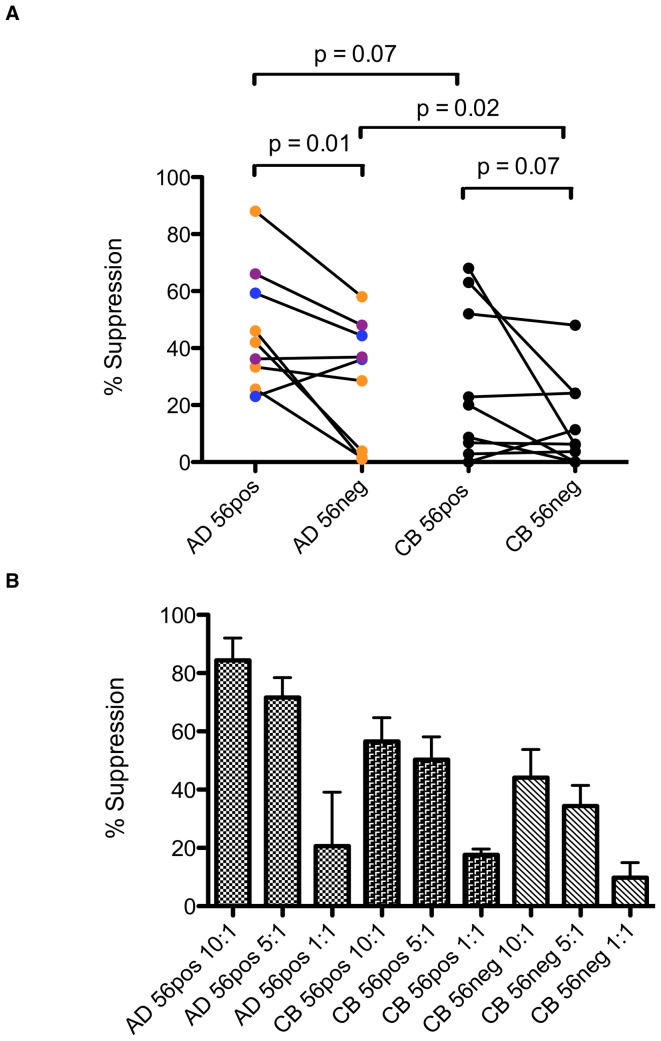
NK Cell-Mediated Suppression of Viral Replication using Autologous HIV-Infected CD4 Lymphocytes. CD56posCD16pos (56pos) or CD56negCD16pos (56neg) NK cell populations from cord blood (CB) (n = 9) or healthy adult donors (AD) (n = 3, repeated in up to 5 experiments) were sorted and cultured with autologous CD4 T cells infected with HIV *in vitro*. Viral replication was assessed by production of HIV p24 antigen and suppression calculated by comparison with the quantity of p24 produced from control infected CD4 T cells without NK effectors. (A) Individual experiments were performed using NK cells at effector : target (E∶T) ratios of 5∶1 from single adult and cord blood samples. Individual adult donors are denoted by color. Generalized estimating equations were used with an exchangeable correlation matrix to account for repeat measures from the same subjects. (B) Representative experiment showing HIV suppression by NK cells at E∶T ratios of 10∶1, 5∶1 and 1∶1 using CD4 and NK cells from a single adult and a single cord blood donor. Data are shown for effectors from adult CD56pos, and cord blood CD56pos and CD56neg NK subpopulations. Histograms depict mean + SEM for sample replicates.

Suppression assays using CD56pos and CD56neg NK cell subpopulations from healthy adults both suggested greater capacity to suppress viral replication in autologous T-cells compared with NK subpopulations from healthy neonates, as depicted at E∶T ratios of 5∶1 in Figure 4A (p = 0.07, 0.02 for CD56pos & CD56neg respectively). Suppression was effector concentration dependent, as NK cells suppressed HIV replication more effectively as E∶T ratios increased from 1∶1 to 10∶1 (Figure 4B). CD56pos NK cell populations from adults and neonates tended to suppress viral replication better than the respective CD56neg populations, although these differences reached statistical significance only among adult donors in the experiments performed (p = 0.01, 0.07 for adult and cord blood respectively). The data shown in Figure 4 show suppression of replication of CCR5-tropic CSF_JR_ HIV, but similar results were observed with the CXCR4-tropic virus HIV-LAI (data not shown).

### Neonatal NK Cells Produce Decreased Anti-Viral Factors in Response to HIV-Infected Cells

**Figure 5:**
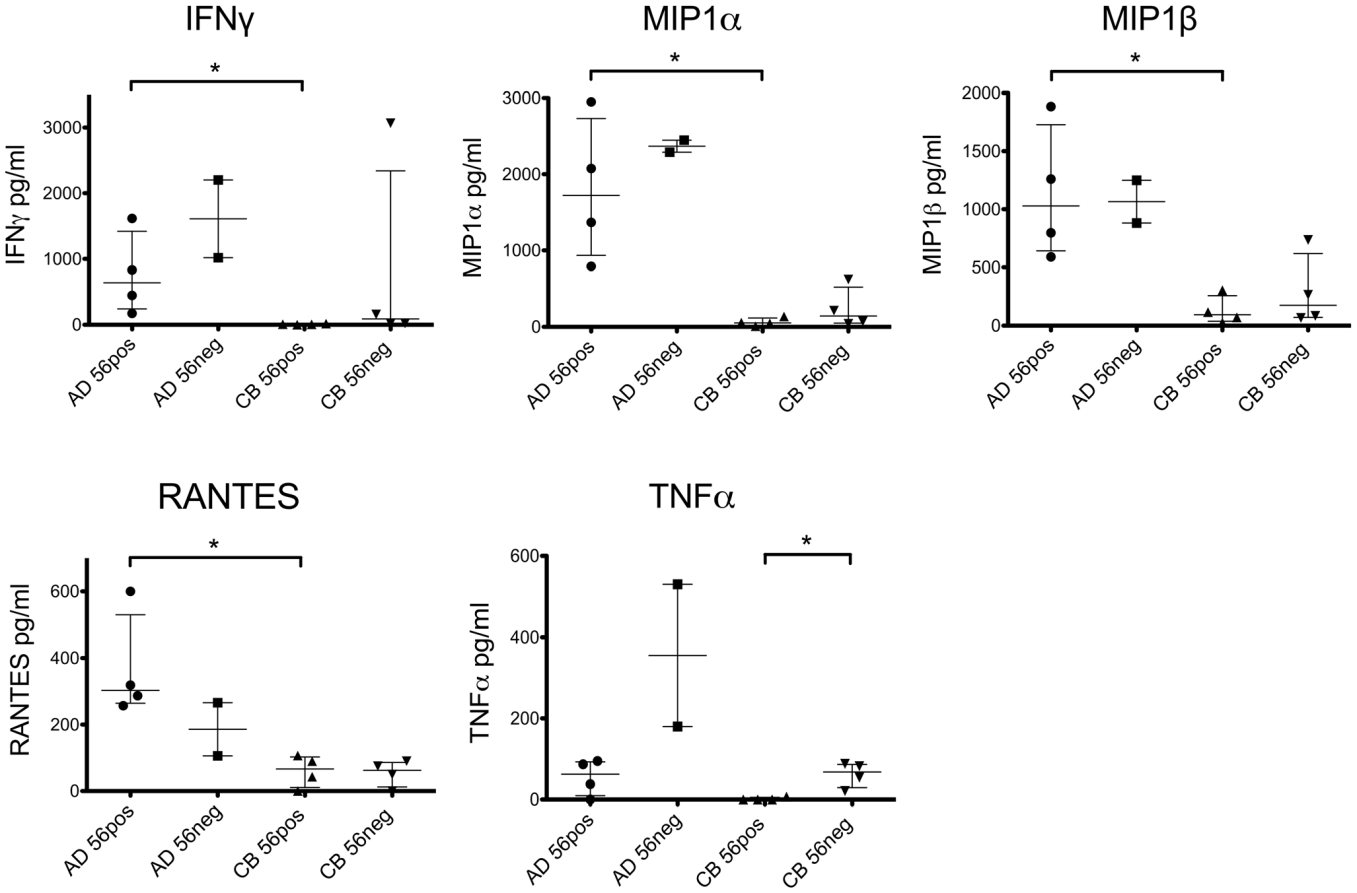
Cytokine Production by NK Cell Sub-Populations from Adults and Infants in Response to HIV-Infected Lymphocytes. CD56posCD16pos (56pos) or CD56negCD16pos (56neg) NK cell populations were isolated from cord blood (CB) (n = 4) and from healthy adult donors (AD) (56pos n = 4, 56neg n = 2). NK cell cytokine production in response to HIV-infected autologous CD4 T cells was assessed from supernatants on day 3 of the *in vitro* viral suppression assay using an 18-plex Luminex assay. Data are shown from 4 individual viral suppression experiments with assays performed in at least duplicate. On two occasions, insufficient cells were obtained from the adult CD56neg sample to allow analysis in replicate. Data are available therefore from four adult subjects for CD56pos effectors but from only two for AD CD56pos cells. Cytokine concentrations (pg/ml) are shown after subtracting ‘background’ values from control HIV-infected CD4 T cells incubated without NK effectors. Bars represent the median and interquartile range. Data for cytokine production were compared between groups using the Mann-Whitney test, * p<0.05. MIP  =  macrophage inflammatory protein; RANTES  =  regulated upon activation, normal T cell expressed and secreted.

The viral suppression assay examines the ability of effector cells to suppress viral replication without identifying the mechanism(s) for suppression. NK cell anti-viral effects may be contributed to by cytolytic activity or by the production of soluble cytokines. *In vitro* cytokine and chemokine production in response to HIV-infection was therefore assessed in supernatants from a number of the viral suppression assays described above. Of the soluble factors assessed in an 18-plex Luminex assay, significant differences between NK fractions were noted in the production of five cytokines from adult and neonatal cell culture (Figure 5). Neonatal NK cells from CD56pos populations produced reduced quantities of the anti-viral cytokines IFNγ, MIP (macrophage inflammatory protein) 1α, MIP-1β and RANTES (regulated upon activation, normal T cell expressed and secreted) compared to healthy adult NK CD56pos cells (p values  = 0.03 for IFNγ, RANTES, MIP1α and MIP1β, Figure 5). CD56neg NK cells from neonates produced more TNF than CD56pos cells (p = 0.03, Figure 5). Differences in cytokine production by AD56neg NK cells may not have been identified in view of the small number of these individuals studied. Other cytokines were not produced in significantly different quantities by the NK cells examined in these experiments.

## Discussion

NK cells isolated from cord blood of healthy infants demonstrated impaired *in vitro* suppression of HIV replication compared with effectors from healthy adults in this study. A reduction in functional NK responses to HIV-infected autologous CD4 cells was accompanied by decreased cytokine and chemokine production and by diminished granzyme B expression in NK cells from newborn infants.

In the only other published study [27] describing NK cell suppression of viral replication in autologous cells infected with HIV *in vitro*, cord blood and adult NK cells both showed effective suppression against a CCR5-tropic virus, but poor antiviral activity against a CXCR4-utlizing virus. In the Bernstein study [27] neonatal NK cells seemed to perform better in inhibiting viral reverse transcriptase production than cells derived from adult donors. Viral replication was assessed and NK cells identified, isolated and stained using different techniques from those employed in our study, but it is not clear why the results reported from that study and our current investigation are different. The β-chemokines MIP1α (CCL3), MIP1β (CCL4) and RANTES (CCL5) are ligands for the CCR5 co-receptor utilized by commonly-transmitted HIV strains, and chemokine production by NK cells has been associated with inhibition of HIV cell entry and replication [32], [33]. Interestingly, NK cells from newborns and adults in the Bernstein study were able to synthesize comparable amounts of the chemokines MIP1α, MIP1β and RANTES on stimulation with IL-2, in contrast to our study where cord blood production of these cytokines was significantly reduced on challenge with autologous HIV-infected cells.

Neonatal NK cells described in this study generally expressed higher levels of both activating and inhibitory NK receptors compared to adult blood, although these differences were largely restricted to the CD56pos subpopulation. Other investigators have also reported increased expression of the activating cytotoxic receptors NKp30 and NKp46 among bulk populations of NK cells in the newborn, together with low levels of inhibitory LIR/ILT2 expression in cord blood cells [29], [30]. The expression of NKp30 and NKp46 are reported to be down-regulated in viremic HIV-infected adults [22] [23], with the loss of Siglec-7 receptor expression particularly apparent in the CD56neg subpopulation in one [31] but not all [23] of these studies. There are few published reports describing Siglec-7 expression on NK cells from cord blood.

Other studies of neonatal NK cell function have typically reported reductions in cord blood NK cell cytotoxic responses to non-physiologic targets including MCH-deficient K562 cells [34] together with impaired cytokine production following in-vitro stimulation of NK cells from newborn infants [8]–[10]. Similar functional impairments have been reported in NK cells from HIV-viremic adults [35], [36], in whom an expansion of CD56neg cells have consistently been identified [21]–[23], although the origins and functional characteristics of this aberrant population of NK cells are currently poorly understood. Our study also identifies an expanded population of CD56neg NK cells in cord blood, and demonstrates for the first time, functional impairment in the response of this NK subset to HIV-infected autologous CD4 cells. CD56neg NK cells from cord blood and from HIV-infected adults in our study had lower expression of the surface receptors Siglec-7, NKp30 and NKp46 compared with their CD56-positive counterparts. By contrast, in a study focusing on recovering NK subpopulations following cord blood transplantation, Della Chiesa et al. report normal levels of Siglec-7 expression among CD56neg NK cells, together with similar levels of NKp30and NKp46 among CD56neg and CD56pos NK cells from donor cord blood [49].

It is unclear whether CD56neg cells identified in cord blood and in other settings represent a common population, or to what extent they might acquire distinct characteristics over the course of chronic viral infection [19], [23], [37] or following hematopoietic transplantation [24], [25]. Of interest, the CD56neg cells described in this study, whether from healthy adult blood, cord blood or HIV-infected blood, showed similar patterns of activating/inhibitory receptors, suggesting that this subset may represent an immature population of NK cells rather than a dysfunctional subset, even in HIV-infected individuals.

The factors that explain the impaired NK effector function found in the newborn period are not well understood, but the observation that disturbances of NK cell function are recognized in other settings where the CD56neg subpopulation is expanded is intriguing. Other potential explanations for neonatal NK cell dysfunction include the possibility that a reduction in the proportion of circulating CD16neg (CD56‘bright’) NK cells, shown here to be reduced in cord blood, may be as or more important than an increase in the CD56neg subpopulation. This NK cell subtype is more widely distributed in secondary lymphoid tissue compared to peripheral blood and these cells may be influential as the predominant manufacturers of NK-derived cytokines [13].

It is increasingly clear that NK cells have an important role not only as cytotoxic effectors but in shaping adaptive responses to intracellular infection [1], [2], [38]. This role appears to be mediated in part through critical interactions with dendritic cells (DC) [39]–[41]. Such interactions may be particularly important in the early response to HIV infection, influencing the quality of antigen presentation to T-cells and shaping the adaptive cellular response in the control of infection [42], [43]. Both dendritic and NK cell function are notably impaired in infants [8], [34], [44], [45], and abnormal NK-DC interactions may contribute to the poor control of HIV-infection and to the rapid progression characteristic of infants infected through mother-to-child transmission [6], [7]. Functional impairments identified in the newborn NK cell population may be influenced by the frequency of CD56neg cells among cellular effectors, and studies of CD56neg NK cells in other settings have identified potentially important disturbances in the interaction between DC and CD56neg NK cells [46].

The origins of the CD56neg NK cell population and the underlying mechanisms of NK cell dysfunction are poorly understood. CD56neg NK cells have been described as ‘anergic’ or ‘exhausted’ in the settings of HIV infection [14] and EBV-driven post-transplant lymphoproliferative disorder (PTLD) [37], but it is possible that impaired functional responses reflect a failure of NK cell development and/or inadequate stimulation in these diverse environments, and may therefore be potentially reversible. Dendritic cells are key determinants of normal NK cell maturation and development, and are an important source of IL-12, known to be required for NK cell activation [40], [47]. Dendritic cells have been shown to produce sub-normal quantities of IL-12 and other cytokines in normal newborns [45] and in HIV-infected individuals [46], [48]. Exposure to IL-2, IL-12 and IL-15 improves cytotoxic effector responses in CD56neg NK cells from newborn infants [11], [16]. It is possible that manipulation of the developmental environment might enable changes in NK cell function and improve the ability to control HIV and other infections in the newborn period. Such interventions have important potential to reduce the risks of transmission and to influence rates of progression in established HIV infection.

## Supporting Information

Figure S1 **Expression of NK Receptors among NK Cell Subpopulations from Cord Blood, HIV-Infected and Healthy Adults.** Surface NK markers were assessed by flow cytometry for CD56pos and CD56neg NK cell subpopulations from healthy adult HIV-seronegative donors, cord blood and chronically viremic HIV-infected adults. A) Representative plots showing NK receptor vs. CD56 expression for CD56posCD16pos (CD56+CD16+) and CD56negCD16pos (CD56-CD16+) NK cell subpopulations from adult, cord blood and HIV-infected adult donors (HIV). B) Fluorescence Minus One (FMO) plots employed in defining thresholds for positivity and for generating the NK receptor expression data shown in Figure 3.
(TIFF)
