# Supplementary figures and images for: Healthy Neonates Possess a CD56-Negative NK Cell Population with Reduced Anti-Viral Activity
Source: PLoS One. 2013 Jun 21;8(6):e67700. doi: 10.1371/journal.pone.0067700 (PMC3689709; doi:10.1371/journal.pone.0067700)

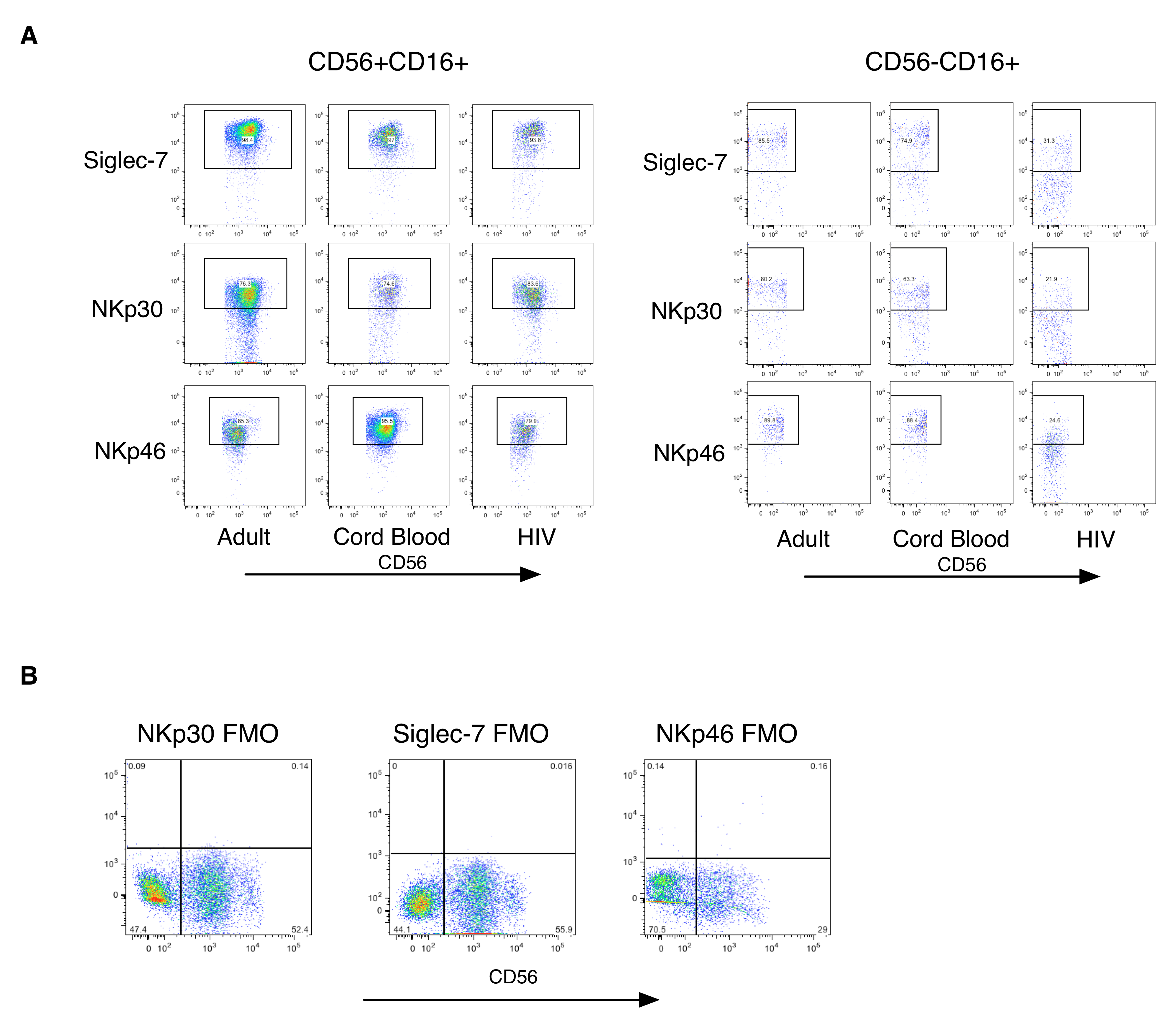

Supplement: Figure S1 — Expression of NK Receptors among NK Cell Subpopulations from Cord Blood, HIV-Infected and Healthy Adults. Surface NK markers were assessed by flow cytometry for CD56pos and CD56neg NK cell subpopulations from healthy adult HIV-seronegative donors, cord blood and chronically viremic HIV-infected adults. A) Representative plots showing NK receptor vs. CD56 expression for CD56posCD16pos (CD56+CD16+) and CD56negCD16pos (CD56-CD16+) NK cell subpopulations from adult, cord blood and HIV-infected adult donors (HIV). B) Fluorescence Minus One (FMO) plots employed in defining thresholds for positivity and for generating the NK receptor expression data shown in Figure 3. (TIFF) [file pone.0067700.s001.tiff]
